# A survey of systemic lupus erythematosus patients' attitudes toward influenza and pneumococcal vaccination in Southwest China

**DOI:** 10.3389/fpubh.2022.1018899

**Published:** 2022-12-20

**Authors:** Yanling Chen, Bo Chen, Xiaolin Shen, Aiping Zhou, Yan Liang, Ying Wang, Hong Chen

**Affiliations:** ^1^West China School of Nursing, Department of Rheumatology and Immunology, West China Hospital, Sichuan University, Chengdu, China; ^2^Department of Rheumatology and Immunology, West China Hospital, Sichuan University, Chengdu, China; ^3^West China School of Nursing, West China Hospital, Sichuan University, Chengdu, China

**Keywords:** vaccination, influenza, pneumococcus, infectious diseases, systemic lupus erythematosus, questionnaire

## Abstract

**Introduction:**

Vaccination is the most effective measure for prevention against infectious diseases in patients with systemic lupus erythematosus (SLE). Therefore, it is important to know SLE patients' attitudes toward influenza and pneumococcal vaccination. This study aimed to investigate the attitude toward influenza and pneumococcal vaccination among SLE patients in Southwest China and its influencing factors.

**Methods:**

A web-based questionnaire was conducted to collect data regarding SLE patients' demographics, history of infections, medications, comorbidities, attitudes toward infection and vaccination, rates of influenza and pneumococcal vaccination, and role of health professionals in promoting vaccination. Univariate and multivariate logistic regression analyses were conducted to assess the vaccination willingness-associated factors.

**Results:**

A total of 251 patients participated in the survey and 240 questionnaires were completed and statistically analyzed. The influenza and pneumococcal vaccination rates were 8.3 and 1.7%, respectively. The top three reasons for non-vaccination were worrying about the SLE exacerbation or flare resulting from the vaccine or its adjuvants, being concerned about adverse events, and the lack of awareness of vaccine availability. More than half of the participants were willing to be vaccinated against influenza (56.2%) and pneumococcus (52.9%). Factors associated to the willingness to receive the influenza vaccine and pneumococcal vaccine were being afraid of infection, believing in the efficacy of influenza vaccination, lower family income, less perceived care from family members, perceived susceptibility to pneumococcal infection, and perceiving influenza and pneumococcal vaccination as beneficial for health.

**Conclusions:**

The influenza and pneumococcal vaccination rates are low among SLE patients in Southwest China. The positive perspective of vaccination on health represented the most impacting factor on their willingness to undergo influenza and pneumococcal vaccination. Non-vaccinated patients were mainly concerned about exacerbation of the disease or adverse events caused by vaccines. It is important to improve the compliance with the guideline-recommended roles of health professionals and to promote the collaboration between rheumatology and primary care teams.

## Introduction

Systemic lupus erythematosus (SLE) represents a heterogeneous systemic autoimmune disease, which involves multiple organs and systems. Patients with SLE have a higher risk of infectious diseases and infection-related morbidity and mortality due to their aberrant immune system, comorbidities and the use of immunosuppressive therapy, despite the improvement in the management of SLE (1–3). The risks of developing severe infections (defined as infections necessitating hospitalization) and invasive pneumococcal infection in SLE are 2.1 times and 13 times higher in patients with SLE compared with the general population (4, 5). In China, infection is the leading cause of mortality in patients with SLE (6–8).

Patients with rheumatic diseases have a higher risk of morbidity and mortality from vaccine-preventable infections (9). Among the causes of death in patients with SLE, vaccine-preventable infection might be a modifiable cause. Examples include the influenza virus and *Streptococcus pneumoniae*, two vaccine-preventable respiratory pathogens that represent significant causes of morbidity and mortality in SLE (5, 10, 11). Thus, it is essential to provide vaccines against influenza and pneumococcus to patients with SLE. These vaccines have been confirmed to be safe and effective and are strongly recommended by European League Against Rheumatism (EULAR) for the majority of patients with autoimmune inflammatory rheumatic diseases, including SLE, particularly those treated with immunosuppressive therapy (12, 13). Annual inactivated influenza vaccination in a single dose is recommended to SLE patients by Canadian Rheumatology Association (14). Meanwhile, pneumococcal vaccination has been designated among the 20 quality measures in the care of SLE patients (15). While influenza vaccine is recommended to be taken yearly, pneumococcal vaccine is taken by a stepwise vaccination strategy, in which the 13-valent pneumococcal conjugate vaccine (PCV13) is administered followed after 8 weeks by the 23-valent pneumococcal polysaccharide vaccine (PPSV23) #1, and then by PPSV23 #2 after at least 5 years (12). For SLE patients, both influenza and pneumococcal vaccinations have been reported to be efficacious and safe (9, 16).

Despite current recommendations, the vaccination coverage in a few countries is unsatisfactory (16–18). A previous review of 12 cross-sectional studies (2578 patients) showed that the pooled proportion of reported influenza vaccination rates, defined as receiving influenza vaccination within 1 year of the study, was 40.0% (95%CI: 33.7–46.5%) (16). The vaccination coverage for pneumococcus was reported to be 25–60% (17, 18), and only 40% of SLE patients were up-to-date on both vaccines (18).

This suboptimal status of influenza and pneumococcal vaccination rates is associated with many factors. For example, the lack of doctor recommendation, efficacy or safety concerns and lack of time were reported to be the most common reasons for not receiving influenza or pneumococcal vaccines in SLE patients (19). A recent study reported that the rheumatologist's patient volume was the most important predictor of pneumococcal vaccination (20), indicating that doctor's recommendation and awareness of the importance of vaccination in infection prevention are essential to promote pneumococcal vaccination. In addition, the vaccination behavior of patients can also be affected by vaccine hesitancy. Vaccine hesitancy is commonly used to describe those who are unsure about or unwilling to receive recommended vaccination due to concerns and doubts about the vaccines, despite the availability of vaccination services (21). Vaccine hesitancy leads to suboptimal coverage of the recommended vaccines and was identified by the World Health Organization (WHO) as one of the top 10 threats to global health in 2019 (22).

Vaccine-hesitant individuals may change their vaccination attitudes and behaviors. Information on the utilization and attitudes toward influenza and pneumococcal vaccination can be useful to guide implementation strategies to improve the vaccination coverage among SLE patients. However, the attitudes of SLE patients toward vaccination and factors relating to vaccination willingness have not been thoroughly investigated. Therefore, in this work, we conducted a survey to estimate the influenza and pneumococcal vaccination rates in SLE patients in Southwest China. We investigated their attitudes toward vaccination and explored the factors influencing vaccination willingness.

## Materials and methods

### Study design and population

In this cross-sectional study, we conducted a web-based questionnaire among SLE patients. The inclusion criteria were as follows: (1) SLE diagnosed by a rheumatologist, using the 1997 American College of Rheumatology (ACR) classification criteria for SLE (23); (2) no < 18 years old. The exclusion criteria were as follows: (1) difficulty reading or understanding Chinese; (2) severe cognitive impairment; (3) unwillingness to take part in the survey.

This study was conducted according to the principles of the Declaration of Helsinki and was approved by the Ethics Committee of West China Hospital (approval number: 20210046). All participants were informed of the purpose of the survey and provided written informed consent before filling out the questionnaire.

### Data collection

The survey was conducted between August 2021 and November 2021. We started by contacting the organizer of the Sichuan SLE patient club to introduce the objective, methods and requirements of this study. After obtaining approval, we distributed the survey link to the members of the patient club, including the questionnaire, written introduction and informed consent of the study. The study was completely anonymous, and the patients were free to decide whether to participate, and they could withdraw from the study at any time. To guarantee the data completeness, the online questionnaire could not be submitted if there were any missing data.

### Questionnaire

The study tool was a self-administered questionnaire and was developed after a thorough literature review and using the findings of earlier studies. The draft questionnaire was reviewed by three experts (two rheumatologists and one physician) to ensure the accuracy of the questions. We also invited two SLE patients to assess the readability of the questionnaire before distribution. The questionnaire included items pertaining to demographic and socioeconomic characteristics, history of infections, medications, comorbidities, attitudes toward infection and vaccination, history of influenza and pneumococcal vaccination, reasons for non-vaccination and the role of health professionals in promoting vaccination. The choices of the *vaccination willingness* included refuse all, refuse but unsure, refuse some, delay, accept some, accept but unsure and accept all. The first four choices were considered to represent vaccination unwillingness, while the last three choices were considered to represent vaccination willingness. For the *correct answer on vaccination*, the item was “can patients with SLE receive vaccination?”, and the choices included: (1) yes, all kinds of vaccines; (2) yes, some kinds of vaccines (correct answer); (3) no; (4) do not know. The choices of the other items were yes/no or agree/disagree.

### Statistical analysis

Continuous data were presented as the mean ± standard deviation or median and interquartile range, while categorical data were presented as absolute count and relative frequency. The age and duration of illness were categorized into quintiles for further analysis. To identify the predictors for vaccination willingness, we calculated the odds ratios (OR) using logistic regression models with willingness to vaccinate as the outcome and the predictors as covariates. The factors were further analyzed in a logistic regression model if *P* < 0.10 in the univariate analysis. A two-sided *p*-value of < 0.05 was considered statistically significant. We used the SPSS software version 25 (IBM, Chicago, USA) for statistical analysis.

## Results

### Participants' characteristics and vaccination status

A total of 251 patients participated in the survey and 240 questionnaires were completed (Table 1). The median age was 32, and 95.4% of the patients were female. The median duration of disease was 79.5 months. Among the participants, 88.3% reported suffering from infections (62.1% for respiratory infection, 47.9% for cutaneous infection and 54.2% for urinary infection) at least once. Emergency room visits and hospitalization due to infections were reported in 79 (32.9%) and 83 (34.6%) patients, respectively, and the median number of hospitalizations was 2 (IQR = 1–5). All the participants had received immunosuppressants since the diagnosis. Instead of health professionals, media was reported as the primary information source of vaccination. The influenza vaccination rate was 8.3%, and 30% of the vaccinated patients received the influenza vaccine every year. Among those who received the influenza vaccines, 90% approved the efficiency of vaccines, and 70% had the plan to continue with vaccination (Table 2). The pneumococcal vaccination rate was 1.7%, among which 50% approved the efficiency of vaccines, and 75% had the plan to continue with vaccination. No adverse events were reported in either vaccine (Table 2).

**Table 1:** Characteristics of the participants.

**Variables**	
Age, years, median (IQR)	32 (26–41)
Females, *n* (%)	229 (95.4)
Educational level, *n* (%)	
Primary school	11 (4.6)
Middle school	60 (25)
High school	49 (20.4)
College or above	120 (50)
Marital status, *n* (%)	
Unmarried	61 (25.4)
Married	154 (64.2)
Divorced, widowed or separated	25 (10.4)
Disease duration, months, median (IQR)	79.5 (45.3–159)
Family income per month (yuan), *n* (%)	
< 1,000	30 (12.5)
1,000–3,999	117 (48.8)
4,000–6,999	61 (25.4)
7,000–9,999	20 (8.3)
≥10,000	12 (5.0)
Residence, *n* (%)	
Urban	129 (53.8)
Suburban	43 (17.9)
Rural	68 (28.3)
Coresident, *n* (%)	
Living alone	35 (14.6)
Living with strangers	2 (0.8)
Living with friends, colleagues or classmates	14 (5.8)
Living with family members	189 (78.8)
Perceived care from family members, *n* (%)	
None	1 (0.4)
Little	12 (5)
A little	69 (28.7)
Some	95 (39.6)
A lot	63 (26.3)
Current state of health, *n* (%)	
Excellent	25 (10.4)
Good	54 (22.5)
Average	119 (49.6)
Bad	36 (15)
Very bad	6 (2.5)
Comorbidities, *n* (%)	68 (28.3)
History of infection, *n* (%)	212 (88.3)
History of respiratory infection, *n* (%)	149 (62.1)
Emergency room visits due to infection, *n* (%)	79 (32.9)
Hospitalization due to infection, *n* (%)	83 (34.6)
Use of medications since diagnosis, *n* (%)	
Corticosteroids	238 (99.2)
DMARDs	211 (87.9)
Biologics	43 (17.9)
Hydroxychloroquine	220 (91.7)
Having consulted health professionals about vaccination, *n* (%)	149 (62.1)

IQR, interquartile range.

**Table 2:** Vaccination rate and information source about influenza and pneumococcal vaccines in SLE patients.

**Variables**	**Influenza**	**Pneumococcal**
	**Vaccine**,	**Vaccine**,
	***n* (%)**	***n* (%)**
Vaccination rate	20 (8.3)	4 (1.7)
Having heard of vaccination	210 (87.5)	66 (27.5)
Information source of vaccine		
Media	83 (34.6)	33 (13.8)
Community bulletin board or brochures	62 (25.4)	23 (9.6)
Search in Internet	78 (32.5)	21 (8.8)
Health professionals	74 (30.8)	19 (7.9)
Family or friends	77 (32.1)	19 (7.9)
Wardmates	61 (25.4)	6 (2.5)

### Attitudes toward influenza vaccination

A total of 67.9% of the patients considered themselves susceptible to influenza infection, and 93.8% believed that influenza would lead to serious consequences. Among the patients, 77.1% were afraid of being infected with influenza, and 87.5% had heard of the influenza vaccine. Only a few patients affirmed the safety (21.7%) and effectiveness (22.2%) of the influenza vaccine, and 56.2% were willing to receive it. The top three reasons for not receiving the influenza vaccine were worrying about the exacerbation or flare of SLE caused by the vaccine or its adjuvants [98 (40.8%)], being concerned about adverse events [91 (37.9%)] and the lack of awareness of vaccine availability [82 (34.2%)], as listed in Table 3.

**Table 3:** Knowledge and attitudes regarding infections and vaccines in SLE patients.

**Statements**	***n* (%)**
Correct answer on vaccination	122 (50.8)
Susceptible to influenza infection	163 (67.9)
Afraid of being infected with influenza	185 (77.1)
Influenza infection will bring about serious consequences	225 (93.8)
Perceiving influenza vaccine as safe for SLE patients	52 (21.7)
Perceiving influenza vaccine as effective for SLE patients	53(22.1)
Perceiving influenza vaccine as beneficial for health	93 (38.8)
Influenza vaccination willingness	135 (56.3)
Reasons for non-vaccination against influenza (*n* = 220)	
Concerned about SLE exacerbation or flare by the vaccine or its adjuvants	98 (40.8)
Concerned about adverse event	91 (37.9)
Lack of awareness of vaccine availability	82 (34.2)
Concerned about no effectiveness	47 (19.6)
Concerned about causing infection	44 (18.3)
Costs	33 (13.8)
People I know are not vaccinated	33 (13.8)
Not recommend by doctors	26 (9.6)
Do not know where and how to get vaccinated	21 (8.8)
Unnecessary	13 (5.4)
Fail in appointment due to lack of vaccines	11 (4.6)
Adverse events relating to vaccination in the past	10 (4.2)
Inconvenient	10 (4.2)
Have no time	6 (2.5)
Susceptible to *Streptococcus pneumoniae* infection	151 (63.0)
Afraid of being infected with *Streptococcus pneumoniae*	201 (83.8)
*Streptococcus pneumoniae* infection will bring about serious consequences	234 (97.5)
Perceiving pneumococcal vaccine as safe for SLE patients	30 (12.5)
Perceiving pneumococcal vaccine as effective for SLE patients	43 (17.9)
Perceiving pneumococcal vaccine as beneficial for health	74 (30.8)
Pneumococcal vaccination willingness	127 (52.9)
Reasons for non-vaccination against *Streptococcus pneumoniae* (*n* = 236)	
Lack of awareness of vaccine availability	127 (52.9)
Concerned about SLE exacerbation or flare by the vaccine or its adjuvants	90 (37.5)
Concerned about adverse events	88 (36.7)
Concerned about causing infection	48 (20.0)
Concerned about no effectiveness	29 (12.1)
People I know are not vaccinated	27 (11.3)
Do not know where and how to get Vaccinated	22 (9.2)
Not recommend by doctors	13 (5.4)
Costs	12 (5.0)
Adverse events relating to vaccination in the past	7 (2.9)
Unnecessary	(2.5)
Inconvenient	3 (1.3)
Have no time	2 (0.8)
Fail in appointment due to lack of vaccines	2 (0.8)

### Attitudes toward pneumococcal vaccination

A total of 63.0% of the patients considered themselves susceptible to *S. pneumoniae* infection, and 97.5% of patients believed that *S. pneumoniae* would result in serious consequences. Among the included patients, 83.8% were afraid of being infected, 27.5% had heard of the pneumococcal vaccine, and 52.9% were willing to receive the vaccine. The top three reasons for not receiving the pneumococcal vaccine were the lack of awareness of vaccine availability [127 (52.9%)], worrying about the exacerbation or flare of SLE by the vaccine or its adjuvants [90 (37.5%)] and being concerned about adverse events [88 (36.7%)], as listed in Table 3.

### The role of health professionals in infection management and vaccination promotion

Only 8.4 and 2.0% of the patients have been recommended to receive influenza and pneumococcal vaccinations by health professionals, respectively. More than half of the participants reported having not received service on infection management from health professionals, such as evaluating the immunization history (65.4%), giving suggestions on the vaccination (82.1%) and explaining matters requiring attention regarding vaccination in SLE patients (73.3%).

### Factors associated with the willingness to vaccination

The following factors were related to the willingness to receive influenza vaccination in the univariate analysis (*P* < 0.10): marital status, comorbidities, correct answer on vaccination, susceptibility to infection, being afraid of infection, being concerned that infection will lead to serious consequences, safety of influenza vaccine, efficacy of influenza vaccine, considering vaccination beneficial for health, suggestions on vaccination by health professionals, and recommendation for influenza vaccination by health professionals. Meanwhile, the following factors were associated with willingness to receive pneumococcal vaccination in the univariate analysis (*P* < 0.10): family income per month, perceived care from family members, correct answer on vaccination, susceptibility to infection, safety of pneumococcal vaccine, efficacy of pneumococcal vaccine and considering vaccination beneficial for health, as listed in Table 4.

**Table 4:** Univariate analysis of factors associated with willingness to get influenza and pneumococcal vaccination among SLE patients.

**Variables**	**Influenza vaccine**		**Pneumococcal vaccine**	
	**OR (95% CI)**	***P***	**OR (95% CI)**	***P***
Age	1.013 (0.844–1.215)	0.893	1.044 (0.870–1.252)	0.643
Gender	0.724 (0.206–2.543)	0.615	0.406 (0.105–1.568)	0.191
Educational level	0.935 (0.779–1.122)	0.935	0.921 (0.791–1.073)	0.292
Marital status				
Married vs. Unmarried Divorced,	2.066 (1.137–3.756)	0.017	1.619 (0.895–2.930)	0.111
Widowed or separated vs. Unmarried	0.867 (0.334–2.250)	0.770	1.027 (0.399–2.646)	0.111
Disease duration	0.925 (0.793–1.078)	0.319	0.929 (0.775–1.113)	0.426
Family income per month	0.836 (0.644–1.085)	0.179	0.788 (0.606–1.024)	0.075
Residence				
Suburban *vs*. Urban	1.422 (0.700–2.889)	0.330	1.064 (0.533–2.125)	0.860
Rural *vs*. Urban	1.068 (0.591–1.928)	0.828	1.104 (0.613–1.991)	0.741
Coresident	1.095 (0.864–1.389)	0.452	1.126 (0.888–1.428)	0.329
Perceived care from family members	0.810 (0.603–1.089)	0.163	0.724 (0.538–0.975)	0.033
Current state of health	0.807 (0.608-1.071)	0.137	0.973 (0.738–1.284)	0.847
Comorbidities	1.632 (0.913–2.916)	0.098	1.284 (0.729–2.261)	0.387
History of infection	1.564 (0.709–3.450)	0.268	1.581 (0.713–3.503)	0.259
Emergency room visits due to infection	1.176 (0.778–1.776)	0.441	1.226 (0.812–1.850)	0.332
Hospitalization due to infection	1.090 (0.767–1.550)	0.631	1.100 (0.776–1.558)	0.593
Having heard of vaccine	0.981 (0.453–2.122)	0.961	1.191 (0.674–2.105)	0.548
Having consulted health professionals about vaccination	1.152 (0.689–1.925)	0.590	1.067 (0.641–1.776)	0.803
Correct answer on vaccination	2.904 (1.712–4.927)	< 0.001	1.766 (1.058–2.946)	0.030
Susceptible to infection	2.230 (1.284–3.871)	0.004	2.594 (1.513–4.446)	0.001
Afraid of being infected	2.135 (1.158–3.935)	0.015	1.564 (0.784–3.122)	0.204
Infection will bring about serious consequences	2.737 (0.906–8.268)	0.074	2.294 (0.412–12.766)	0.343
Perceiving vaccine as safe for SLE patients	10.682 (4.068–28.050)	< 0.001	3.349 (1.378–8.141)	0.008
Perceiving vaccine as effective for SLE patients	20.000 (6.023–66.407)	< 0.001	5.991 (2.543–14.110)	< 0.001
Perceiving vaccination as beneficial for health	15.163 (7.249–31.720)	< 0.001	10.463 (5.010–21.854)	< 0.001
Role of health professionals				
Ask about immunization history	1.024 (0.599–1.750)	0.932	1.006 (0.590–1.714)	0.983
Suggest vaccination to prevent infection	2.022 (0.996–4.106)	0.051	1.637 (0.830–3.226)	0.155
Recommend influenza/pneumococcal vaccine	3.638 (1.186–11.162)	0.024	1.343 (0.220–8.184)	0.749
Explain matters needing attention regarding vaccination in SLE patients	1.555 (0.862–2.806)	0.143	1.309 (0.735–2.333)	0.360

In the multivariate analysis, being afraid of infection with influenza, believing in the efficacy of the vaccine and perceiving influenza vaccine as beneficial for health were associated with the willingness to receive the influenza vaccine (Table 5), while lower family income, less perceived care from family members, perceived susceptibility to pneumococcal infection and perceiving pneumococcal vaccine as beneficial for health were associated with the willingness to receive the pneumococcal vaccine (Table 6).

**Table 5:** Multivariate analysis of factors associated with willingness to get influenza vaccination among SLE patients.

**Variables**	**B**	**S.E**	**Wald**	**OR (95% CI)**	***P***
Afraid of being infected	0.836	0.392	4.554	2.306 (1.071–4.969)	0.033
Perceiving vaccine as effective for SLE patients	1.681	0.687	5.992	5.372 (1.398–20.643)	0.014
Perceiving influenza vaccination as beneficial for health	2.068	0.422	24.044	7.909 (3.460–18.076)	< 0.001
Constant	−1.280	0.360

**Table 6:** Multivariate analysis of factors associated with willingness to get pneumococcal vaccination among SLE patients.

**Variables**	**B**	**S.E**	**Wald**	**OR (95% CI)**	***P***
Family income per month	−0.331	0.164	4.087	0.718 (0.521–0.990)	0.043
Perceived care from family members	−0.419	0.182	5.287	0.658 (0.460–0.940)	0.021
Susceptible to infection	1.123	0.332	11.423	3.073 (1.603–5.893)	0.001
Perceiving pneumococcal vaccination as beneficial for health	2.688	0.417	41.545	14.703 (6.493–33.296)	< 0.001
Constant	1.145	0.883

## Discussion

There were three major findings arising from our study. First, we found that the influenza and pneumococcal vaccine coverage were suboptimal among SLE patients in Southwest China. Second, the main reasons for non-vaccination were worrying about the SLE exacerbation or flare resulting from the vaccine or its adjuvants, being concerned about adverse events and the lack of awareness of vaccine availability. Third, the most important factor associated with patients' willingness to receive the influenza or pneumococcal vaccine was perceiving influenza or pneumococcal vaccination as beneficial for health. Finally, only a small proportion of health professionals provided vaccination-related assessment, recommendation, and education.

In this study, all the participants had received immunosuppressants since the diagnosis, but the vaccination coverage of influenza and pneumococcus was low. A small proportion (8.3%) of the participants had taken the influenza vaccine, and only 6 patients declared receiving the influenza vaccine every year, which is proposed by the guidelines. Only 1.7% of the participants had received pneumococcal vaccination. Overall, the vaccination coverage of influenza and pneumococcal among the SLE patients in our study was much lower than that reported in other studies. A systemic review (16) revealed that the pooled proportion of influenza vaccination within 1 year was 40.0%, while the pooled ever-vaccination rate was 60.2%. In an intervention study (20), the baseline PCV13 vaccination rate, PPSV23 vaccination rate and combination vaccination rate were reported to be 2, 8, and 10%, respectively. The coverage of pneumococcal vaccines was 25, 32.2, and 32.8% among SLE patients from Latin America (17), Germany (24) and the United States (19). However, the results of our study are higher than those of a previous study conducted in Southern China in 2017. Jiang et al. reported 0.4% and 0% vaccination rates for influenza and pneumococcal vaccines, respectively (25). This may be explained by the outbreak of COVID-19, which has raised awareness of vaccination among the public, and the promotion of chronic disease management in the Chinese rheumatology field. In fact, more than half of the participants in our study claimed that they had consulted health professionals about vaccination. Chronic disease management is a transdisciplinary care model, in which rheumatologists with allied health professionals (e.g., nurses, psychologists, pharmacists) provide consultation, regular monitoring, risk assessment and comprehensive interventions to delay the disease progress, reduce complications, maintain the functionality, improve the quality of life (QoL) and reduce medical costs. This care model has been adopted by an increasing number of health professionals in mainland China, giving more SLE patients access to health education and consultation provided by rheumatology nurses.

Our findings showed that there were complicated reasons why SLE patients did not receive the vaccines recommended by the guidelines. The main reason for not receiving influenza vaccine was concerns about its safety. Other reasons included worrying about the possibility of SLE exacerbation or flare by the vaccine of its adjuvants and adverse events as well as the lack of awareness of vaccines that can be received by SLE patients. The proportion of patients with rheumatic diseases who thought influenza vaccine would worsen their disease and listed it as a reason for non-vaccination was higher in our study compared with previous studies (26, 27). A meta-analysis reported that the most common reasons for influenza non-vaccination were the lack of doctor recommendation or medical prescription (57.4%) as well as concerns over the efficacy or safety of the vaccine (12.7%) (16), which is a little different from our study. Among our participants, only 9.6% listed the lack of doctor recommendation as a reason for non-vaccination. Our results illustrated that providing information about vaccine safety is more needed than direct recommendations. As for the pneumococcal vaccine, the lack of awareness of the vaccine that can be received by SLE patients was listed as the main reason for not taking the pneumococcal vaccine. The most common reason cited by patients who did not receive PPSV23 was “not recommended” (72%), followed by “no reason given” (24%) and “do not like shots” (4%) (28). These were similar to what was reported in another study: lack of recommendation (87%), lack of time (7%) and efficacy or safety concerns (4%) (16). These concerns should be taken seriously, as they can influence the vaccination behavior of SLE patients.

Influenza vaccine and PPSV23 are optional vaccines and not free in China. Therefore, patients with autoimmune inflammatory rheumatic diseases or under immunosuppressive treatment undergoing vaccination need pay the bill by themselves. But the cost can be reimbursed by medical insurance. The cost was reported as a public barrier to vaccination (29). In our study, the percentage of nonvaccinated patients who listed cost as the reason for not receiving the vaccine was 15% for influenza vaccine and 5.1% for the pneumococcal vaccine. Our findings were consistent with two other studies in China (24, 30), which indicates that cost was less concerning than other factors.

Our regression analysis showed that SLE patients with a higher willingness to receive the influenza vaccine were more likely to be afraid of influenza infection, believe in the efficacy of the vaccine and consider getting the influenza vaccine to be beneficial for their health. According to a study on patients with rheumatic disease, vaccination barriers included perceptions that infections would not be serious problems and that they would not benefit from vaccination (25). Another study showed that concerns about the efficacy of the influenza vaccine were more prevalent in rheumatic disease patients who had never been vaccinated against influenza (26). These results illustrated emphasizing the risk of influenza infection and positive aspects of influenza vaccination to be essential for promoting vaccination willingness. In our study, lower family income, less perceived care from family members, perceived susceptibility to pneumococcus infection and perceiving the pneumococcal vaccine as beneficial for health were predictors for the willingness to receive the pneumococcal vaccine. Patients might be more independent if they lack the economical and/or mental support from family, which makes them pay more attention to their health. A study on COVID-19 vaccination also found the probability of vaccine hesitancy among the high-income population to be higher than that in the low-income population (31). This may be because high-income people have more resources to create better living environments and obtain more personal protective equipment, so they feel that good protection can replace vaccination (31). However, considering the different features between pneumococcal and vaccination and the freely provided and better known COVID-19 vaccination, the impact of socioeconomic factors on the vaccination willingness should be further investigated. Factors influencing the acceptance of recommended vaccines include the individuals' perception of their susceptibility to diseases and the severity of vaccine-preventable diseases along with vaccine safety and efficacy concerns (19, 21, 32). We found that SLE patients were more concerned about the positive effects of the vaccination, which indicates that education emphasized the efficacy of vaccines.

The active involvement of health professionals is critical for infection management and vaccination promotion. It is suggested that the vaccination status and indications for further vaccination should be assessed yearly by the rheumatology team (12). However, our findings showed that only 34.6% of the participants had been asked about the vaccination status by health professionals, and the proportion of patients who were assessed by a rheumatologist, delegated for vaccination status assessment, was 26.3%. It has been demonstrated that better knowledge about vaccination and its recommendation by a treating specialist were positively associated with an improved vaccination rate among patients with autoimmune inflammatory rheumatic diseases (33, 34). Nevertheless, only a small number of participants (8.8% for influenza and 2.1% for pneumococcus) in our study declared that the vaccine was proposed to them by health professionals, indicating that health professionals were less aware of the importance of vaccination and lack of the initiative in vaccine recommendation, especially the pneumococcal vaccine. Kernder et al. reported that vaccination counseling, as one of the indices of the quality of care, predicted better outcomes in SLE patients (35). Explaining the individualized vaccination programme to the patients by rheumatology team was listed as an overarching principle in the 2019 EULAR recommendations (12). However, our survey found that the main information source about the influenza and pneumococcal vaccines for those who heard of it was the media. As we all know, some of the information from media is not verified by professionals, so there is a possibility that SLE patients may get misled regarding vaccination. Our results indicated the importance of health professionals taking a more active part in the dissemination of information on vaccination among SLE patients through media. Vaccination counseling provided by trained health professionals can be integrated into standard care of SLE patients.

Collaboration between rheumatology and primary care teams is critical to support the implementation of vaccination and maximize its rate. In China, all vaccinations are undertaken in primary care. Thus, the physician's awareness of the necessity of vaccination and capability to complete assessment and recommendation are critical to promote vaccination in SLE patients. However, most of the primary care staff have insufficient knowledge on the management of SLE and have difficulty proposing suggestions on vaccination. A recent study found that the volume of SLE patients seen by rheumatologists is strongly associated with the likelihood that the patients will have PPSV23 recommended and delivered (28). Rheumatologists usually have a larger SLE patient volume than physicians, which makes the former more experienced with the management of SLE complexities and more sensitive to the importance of preventive care (28). The rheumatology team and primary care team can cooperate in assessing the risk of infection, identifying the indications for vaccinations and informing the patients about the risk/benefit ratio of vaccines. Meanwhile, training programmes regarding infection management of patients with autoimmune inflammatory rheumatic diseases should be developed for primary care health professionals. Moreover, decision aids and electronic alert systems can be developed to promote assessing the vaccination need of SLE patients and recommending vaccination for eligible ones.

Our study has some limitations. First, the data were self-reported and therefore susceptible to recall bias. Second, we included SLE patients *via* a web-based approach, which could result in a selection bias that only the patients interested in vaccination took part in the survey. In addition, we only included the patients who had joined a patient club in Southwest China, which was not representative of the population of SLE patients in China. Finally, the sample size was small, and it is possible that many of the negative findings were due to insufficient numbers to detect the differences between groups.

In conclusion, the coverage rates of influenza and pneumococcal vaccination were both low among SLE patients in Southwest China. The positive perspective of vaccination on health represented the most effective factor on the willingness to receive influenza and pneumococcal vaccines, which indicates the significance of providing detailed and convincing information on the health benefits of vaccines. Our findings also suggest a growing attention toward vaccination in SLE patients and the importance of cooperation between rheumatology specialists and primary healthcare physicians both in the management of infection and educating patients regarding the risk of infection, effectiveness and benefits of vaccination.

## Data availability statement

The raw data supporting the conclusions of this article will be made available by the authors, without undue reservation.

## Ethics statement

The studies involving human participants were reviewed and approved by the Institutional Review Board of West China Hospital, Sichuan University and conducted in accordance with the Declaration of Helsinki (revised in 2013). The patients/participants provided their written informed consent to participate in this study.

## Author contributions

YC and HC designed the study and completed statistical analysis. YC, XS, AZ, YL, and YW distributed and collected the questionnaires. YC performed statistical analysis and drafted the manuscript. BC and HC interpreted of data and refined the manuscript. All authors contributed to the study conception, design, and approved the final manuscript.
